# An Atypically Large, Free-Floating Thrombus Extending From the Lung to the Left Atrium via a Pulmonary Vein: A Case Report

**DOI:** 10.1097/MD.0000000000001853

**Published:** 2015-11-20

**Authors:** Wei Wang, Xuechang Li, Weian Song, Yunshan Zhang, Caiying Yue, Liqun Shang, Jun Li, Feng Wen, Junqiang Liu, Peng Zha

**Affiliations:** From the Department of Thoracic Surgery (WW, XL, WS, CY, LS, JL, FW, JL, PZ) and Department of Ultrasound (YZ), PLA Navy General Hospital, Beijing, China.

## Abstract

An atypically large, free-floating thrombus extending from primary pulmonary malignancy into the left atrium (LA) is a rare phenomenon. Here, we report a 61-year-old man presenting with a large mass in the lower lobe of the left lung, extending to LA via the left inferior pulmonary vein.

The thrombus remained clinically silent and was detected by computed tomography (CT) and transthoracic echocardiography. To prevent life-threatening complications including systemic embolism and sudden death, the patient underwent surgical excision of the mass under cardiopulmonary bypass. Pathology of the tumor and the embolus was confirmed as moderately differentiated squamous cell carcinoma. Furthermore, immunohistochemical studies demonstrated consistency of the tumor cells in this pathological category.

The patient tolerated the surgery well and his condition began to improve gradually after the operation.

## INTRODUCTION

Incidence of cardiac metastases has increased over the last 30 years. Perhaps owing to increased life expectancy of oncology patients benefitting from advances in cancer diagnosis and management.^1^ The following case report describes a 61-year-old man with an atypically large, free-floating tumor embolus extending to the left atrium (LA) via the left inferior pulmonary vein. Though there are no standard treatments available for this particular patient, surgery should be chosen as first line of intervention for large embolus formation in the LA.

## MATERIALS AND METHODS

### Case Report

**FIGURE 1:**
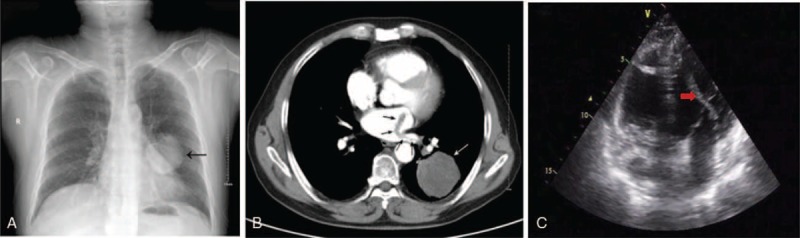
A chest radiograph (A) and computed tomography scan (B) showed a mass of 5.5 cm in the left lower lobe and a linear band of 2.0 cm behind the LA (arrows). Transthoracic echocardiography (C) displayed 8 to 10 cm embolus extension (red arrow), highly mobile, from the LA prolapsing to the left ventricle. LA = left atrium.

A 61-year-old man presented with a low-density, round-shaped mass with well-defined margins measuring 5.5 cm on his preoperative chest radiograph (Fig. 1A). A computed tomography (CT) scan of the chest revealed a mass measuring 6.0 × 5.0 cm in the lower lobe of the left lung and a linear band of 2.0 cm behind the LA (Fig. 1B). Transthoracic echocardiography (TTE) showed a thin, linear hypoechoic band of 0.9 cm in the left inferior pulmonary vein ostium and also revealed a highly mobile embolus extension 8 to 10 cm in length protruding into the LA (Fig. 1C). Since the extension prolapsed into the left ventricle toward the mitral valve in diastole and into the LA with the retrograde motion of systole, a small amount of blood flowed backward past mitral orifice due to the embolus-leading mitral regurgitation. There was no evidence of enlargement or injury of the LA. This study was approved by the Institutional Review Board of PLA Navy General Hospital. The patient has signed a written informed consent form.

**FIGURE 2:**
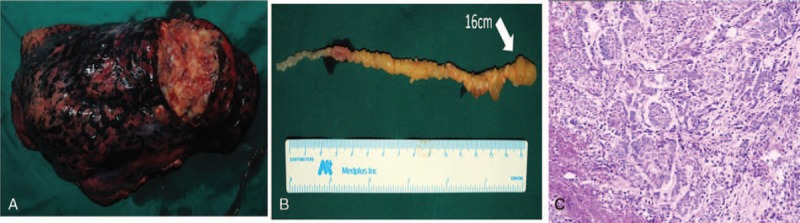
A heterogeneous mass (3.0 × 9.5 × 6.0 cm) of solid and cystic components with areas of hemorrhage and necrosis was seen in a gross architecture of the mass (A). A giant, free-floating embolus (16.0 × 0.9 cm) consists of multiple grape-like clusters of soft tissue pedunculated on a stalk (B). The microscopic finding (H&E stain, ×100) demonstrated as moderately differentiated squamous cell carcinoma (C).

A left anterolateral thoracotomy was carried out under general anesthesia. After the lung mass was isolated from the lower lobe of the left lung, the veins, arteries, and bronchial tube to the area of the mass were cut and the mass was removed from the left lower lobe (Fig. 2A). After which, the blood vessels and bronchial tube was sutured and the patient was checked for any bleeding in and around the lungs. Following lobectomy, embolectomy was executed under cardiopulmonary bypass. A free-floating embolus of 16 cm in length was removed from the LA together with the excision of the embolus extending up to the left inferior pulmonary vein around its base. A grape-like cluster of soft tissue was observed in the extension (Fig. 2B). Pathological examination of the lung mass showed spindle-shaped fascicular proliferation with nuclear palisading (Fig. 2C) indicating moderately differentiated squamous cell carcinoma (SCC). Immunohistochemistry was used to confirm SCC. The cells from the lung tumor and the embolus extension showed cytokeratin (CK) AE1/AE3 (+++) and p63 (+++). The patient recovered uneventfully and was discharged on postoperative day 15.

## RESULTS AND DISCUSSION

Though tumor emboli extending into LA are not uncommon,^2–4^ such a large embolus is extremely rare in primary nonsmall cell lung cancer (NSCLC) metastasis. It has been found that the frequency of cardiac metastases varies from 12% to 25% in postmortem patients who have died of malignancies and lung tumors show the most cardiac metastatic potential in the common tumors.^5,6^ In patients such as the 1 described in this study who are completely asymptomatic, an early diagnosis of the lung tumor with the embolus extending into the LA can be challenging. CT scan and pathological examination of the lung mass would be additional tools used for cardiac imaging during the differential diagnosis of the LA mass taking into consideration that 36% to 49% of cardiac metastasis may occur in NSCLC patients.^7,8^ In contrast to CT imaging, TTE is more accurate in the diagnostic process of the patient because this technique may display moving images of the heart particularly for the evaluation of a cardiac mass.

As the patient was considered to be at risk of systemic embolism and sudden cardiac dysfunction due to the intracardiac embolus,^4,9^ left anterolateral thoracotomy was selected in this case. Since the removal of the embolus could associate with severe bleeding in the presence of dense adhesions, cardiopulmonary bypass was applied to ensure hemodynamic stability during surgery. Embolectomy was successfully performed despite the atypical size. This was facilitated by performing an inverted T-shaped atrial incision by which a line of cleavage was identified between the embolus and left atrial wall. Additionally, disconnection of the left inferior pulmonary vein was required when the whole embolus was completely removed in the surgical process. This not only shortened surgical time but also reduced risk of embolic event due to incomplete resection of the embolus extension. The presence of dense adhesions and absence of cleavage plane may make embolus removal difficult and the residual organized material of the embolus can be left under these circumstances. In such cases, autologous pericardial patch may be used to cover these areas to avoid future embolus formation.^10^ It is necessary to emphasize that a scheduled surgery should be executed as early as possible because any delay in treatment can be fatal and increase difficulty of the operation. Although safe resection of the large embolus depends largely on the degree of cardiac involvement, this patient tolerated the given surgical therapy well and his condition began to improve gradually after the operation.

Pathological examination of the postoperative patient revealed that the tumor consists of fascicular proliferation with nuclear palisading and a structureless array. Immunoreactivities of the mass and the embolus were all positive for AE1/AE3 (+++) and p63 (+++), which are consistent with a diagnosis of SCC. Postoperative examination of specific CK expression was used to gain valuable information as to the primary site of the tumor embolus. The result of pooled CKAE1/AE3 antibody demonstrated the embolus originated from epithelial carcinoma as the CK cocktail AE1/AE3 distinguishes epithelial carcinoma from nonepithelial malignancies and is often used to characterize the source of various neoplasms.^11^ P63, a well-known marker of SCC differentiation,^12^ was over-expressed in both specimens, indicating consistency of these tumor cells in pathological category.

The size of the embolus was unusually large in comparison to previously reported cases despite the fact that the size of LA in our patient was normal. The exact etiology is not fully understood. It is likely that the hypercoagulable state of oncology patients is a contributing factor to formation of emboli in this size.^13^ It has been reported that some embolic symptoms can constitute the first manifestation of cancer-associated thrombosis.^14^ However, this patient never experienced these clinical events, suggesting that application of prophylactic anticoagulants was not required for him as necessary in preoperative and postoperative treatment because cancer patients may also have a higher risk of bleeding with the anticoagulant therapy.

Better understanding of postoperative patients who will benefit from adjuvant chemotherapy is a field of active investigation.^15,16^ This patient did not receive the adjuvant treatment with reasons including the type of the tumor, the patient's age, and overall health. Since NSCLC respond very poorly to chemotherapy drugs and the therapy itself may also cause unpleasant side effects,^17^ clinicians should individualize their treatment approach to the patient's specific needs.
